# Sports Gamification: Evaluation of Its Impact on Learning Motivation and Performance in Higher Education

**DOI:** 10.3390/ijerph18031267

**Published:** 2021-01-31

**Authors:** Taofeng Liu, Mariusz Lipowski

**Affiliations:** 1 School of Physical Education (Main Campus), Zhengzhou University, Zhengzhou 450001, China; 202033084@sangmyung.kr; 2 Department of Physical Education, Sangmyung University, Seoul 390-711, Korea; 3 Department of Psychology, Gdansk University of Physical Education and Sport, 80-336 Gdansk, Poland

**Keywords:** sports games, motivation, experimental teaching, learning performance, tennis skills

## Abstract

In this study, the impacts of sports gamification on college students’ learning motivation and learning performances were explored by training students majoring in physical education to play tennis. A total of 150 students from a physical education college were selected to participate in this experimental teaching, and they were divided into the experimental group (EG) and the control group (CG). Based on the above purposes, the differences in the teaching methods and teaching objectives of the EG and the CG is that the former uses games as a key method in tennis teaching. All participants were asked to complete questionnaires, with the purpose of evaluating the learning motivation of tennis before and after sports game intervention. Additionally, the differentiated learning motivation and learning performance between EG and CG before and after experimental teaching was tested and evaluated. Results demonstrate that students in the EG have significantly increased their intrinsic motivation and introjected regulation, thereby showing better results than CG in key test items. In addition, the above result reveals the positive role of sports gamification in promoting the learning motivation and performance of college students.

## 1. Introduction

With the deepening of educational reform and development in China, the tasks and requirements of physical education are also increasing. As a result, gamification has received widespread attention in physical education [1,2]. The developmental process of games is similar to that of human social practice activities. Although there is no unified definition of games, games are actually a form of activity [3]. Different from games in other fields, sports games have the most fundamental feature of movement of the limbs, which is a crucial auxiliary method in physical education; besides, sports games are more targeted than other games [4,5]. At present, there is no unified definition of ball games, but from a broad perspective, ball games present ball-related knowledge and skills, with the primary purpose of strengthening physical fitness and developing competitive levels. Physical education is of great significance in improving the physical fitness of students, and sports games are a key component of school physical education. Sports games are interesting and distinctive, with rich types and diverse forms. In the meantime, they depend less on hardware facilities including venues and equipment. Therefore, sports games can help students concentrate, mobilize enthusiasm, and improve physical fitness, thereby playing a positive role in cultivating sports habits. Sports gamification combines sports activities with games. Sports games with regular expressions can be divided into two types: physical development and intellectual development. Apparently, sports games can improve students’ physical fitness and help students promote physical and mental health, develop intelligence, and cultivate flexible logical thinking ability [4,6,7]. In short, sports gamification is a comprehensive skill or method, which has an important value in promoting the overall development of students.

According to the internalization of an individual’s self-determination motivation, motivation can be divided into intrinsic motivation and extrinsic motivation. The former is characterized by the individual’s pleasure or satisfaction in related activities, whereas the latter is characterized by the individual’s motivation to strive for a particular goal [8,9]. As a result, sports motivation directly promotes people to participate in sports activities. The Self Determination Theory (SDT) based on learning motivation not only emphasizes the role of external environmental factors, but also focuses on self-integration. SDT believes that intrinsic motivation, extrinsic motivation, and no motivation are its three primary forms. Extrinsic motivation can be divided into external regulation, identification regulation, integrated regulation, and introjected regulation [10,11,12]. As for the physical education courses, the SDT theory emphasizes that the interaction between people and the environment will produce different motivations, which have an inseparable relationship with people’s specific situations. The physical activities that individuals engage in are affected by multiple factors, such as social environment, physiological and psychological factors. Therefore, integrating sports motivation and training behavior is of great significance [13]. The evaluation and measurement of learner motivation have attracted widespread attention in the game learning field. Ghergulescu and Muntean [14] proposed a real-time and non-interference automatic measurement and analysis method for game learning motivation based on EEG sensors. Taking students who participate in educational games as the research objects, it was found that the learners’ motivational opportunities would change over time during the game. Physical education classes are beneficial to cultivating students’ motivation for lifelong physical education. Luo et al. [15] investigated the influence of team game competitions on students’ learning motivation and motor skills, and the results of pre-test-post-test experimental teaching found that team game competition strategies could significantly improve the learning motivation of students. However, these strategies had little effect on improving motor function. Ability level had no significant influence on motivation but a significant correlation with the acquisition of motor function. Hence, there was a close relationship between sports games and students’ learning motivation.

As for ball games in physical education, the traditional teaching methods mostly adopt class teaching methods, which are often teacher-centered. However, students are in a passive state of acceptance, which is detrimental to the overall development of students. Traditional teaching methods cannot fully consider the psychological needs of students. As a result, new methods are needed to motivate students’ sports learning, and introducing sports games is a useful way to mobilize students’ enthusiasm and initiative. The same applies to tennis training in colleges and universities. In summary, the primary purpose is to explore sports gamification’s impact on college students’ motivation and performance in learning tennis. In this regard, the following hypotheses were proposed in this study:

**Hypotheses 1 (H1).**  *Compared with the control group (CG), the experimental group (EG) has a more significant increase in the intrinsic motivation of tennis learning.*

**Hypotheses 2 (H2).**  *Compared with the control group (CG), in the experimental group (EG), sports games will increase students’ motivation to learn tennis.*

**Hypotheses 3 (H3).**  *Compared with the control group (CG), the experimental group (EG) can obtain better results in learning tennis skills.*

## 2. Materials and Methods 

This experimental teaching was conducted based on the college’s tennis optional course. This course is a vital component in the physical education of colleges and universities. The experimental group (EG) and the control group (CG) are divided for facilitating the experimental teaching and verifying the above hypotheses [16,17]. The entire experimental teaching lasted for a quarter (13 weeks), with two class hours per week. The class time of EG and CG were both 14:00–15:00, and the class time of EG and CG was one day apart. The basic situation of the samples was balanced according to the physical fitness of the participating students to ensure the fairness and justice of the entire experimental teaching. All participants voluntarily participated in the questionnaire survey for the teaching experiment, and fully understood the purpose of the questionnaire survey. The specific implementation plan of the experimental teaching has been verified by experts in the sports field. This teaching experiment will not adversely affect the health, safety, and rights of participants.

### 2.1. Sample Selection

The research objects were students from the tennis-teaching class of Xi’an Physical Education University. The research objects were selected in a random manner. The division of the selected class and the proportion of students all follow general standards. At first, 200 college students were selected in this experimental teaching. According to the exclusion criteria introduced below, 150 participants were finally obtained. The specific gender distribution is shown in Table 1. There were 80 males and 70 females, accounting for 53.33% and 46.67%, respectively. The participants’ average age was 21 ± 3.

In addition, the exclusion criteria are as follows:

(1) The experimental teaching was regular; the entire evaluation process should be uninterrupted; the overall course hours finished should exceed 80%. Those who did not finish the required class hours were excluded (18 in EG and 16 in CG).
(2) Those who did not finish the questionnaire were excluded (8 in EG and 5 in CG).
(3) Those who were injured physically were excluded (1 in EG and 2 in CG). All the participants were clearly aware of the overall process of the experimental teaching, and all the research results were announced anonymously after the participant had signed a confidentiality agreement.

### 2.2. Instruments

#### 2.2.1. Determination of Tennis Learning Motivation

A tennis motivation questionnaire was designed to evaluate the motivation of the participants. This questionnaire was used twice in this experimental teaching before and after sports game intervention. The pre-test questionnaire was implemented before the experimental teaching started, i.e., before dividing the students into CG and EG, which could avoid possible deviations in the samples. Specifically, the questionnaire contained 25 questions, and it was divided into four levels: participation, attention, enthusiasm, and negativity of tennis. These components could evaluate participants’ intrinsic motivation, such as “interested in this sport” and “this sport can improve the skills.” Questions for external influence factors included “the teacher thinks playing tennis is useful.” Motivational variables used for evaluation included active input (introjected regulation) [18], vitality interest (intrinsic motivation) [19], skill mastery (integrated regulation) [20], and external encouragement (external regulation) [21]. The questionnaire used the Likert five scale [22,23] to score. Answers were numbers ranging from 1 to 5, in which 1 represented “strongly disagree” or “strongly dissatisfied”, and 5 represented “strongly agree” or “strongly satisfied”. The validity of the questionnaire was tested by expert evaluation. Among them, 60% of the 10 experts considered the questionnaire to be basically reasonable, 30% considered reasonable, and 10% considered very reasonable. No one thought the questionnaire was unreasonable. It is obvious that the questionnaire designed this time has high validity. The reliability of the questionnaire was tested by the retest method. Results suggest no significant difference between the two evaluation tests. Besides, the retest data are good, and the questionnaire has high reliability.

#### 2.2.2. Evaluation Tests

Students’ performance in learning tennis was evaluated through tests in the last class. This evaluation test included a standard test of tennis skills. The key test items included the serve, the forehand drive, and backhand drive, each totaling ten shots. The evaluation criteria adopted by EG and CG were the same. The participants served twice in the deuce court and advantage court and rotated, totaling ten serves. The participant could score if the served ball were within the effective coverage. The teacher served the ball for the participants while testing the forehand and the backhand drives. The participants completed 20 drives and backhand drives at the midpoint of the baseline. The participant could score if the ball hit the effective coverage. Remarkably, the scoring standard was as follows: Ten serves within the effective coverage would be counted as ten points; by analogy, and 0 serves within the effective area would be 0 points. Similarly, ten drives/backhand drives within the effective coverage would be ten points; by analogy, 0 drives/backhand drives within the effective coverage would be 0 point.

### 2.3. Implementation Process

This experimental teaching lasted for three months, totaling 26 class hours. EG and CG were taught by the same teacher. During the entire experimental teaching process, except for the game link, the two groups were exposed to the same teaching content, the length of the interval between classes, and the completion progress of each stage of the teaching. The testing standards, equipment, and venues used were also the same. Components that could affect the results were strictly controlled during the entire experimental teaching process. The detailed teaching stages and teaching designs are shown in Table 2 below.

The first questionnaire survey was conducted over the participants before the experimental teaching, i.e., the pre-test tennis motivation questionnaire. During this period, all participants would be introduced to the follow-up experimental teaching courses and particular implementation links, so that the participants of EG and CG were aware of the implementation details of the entire experimental teaching process. In the following three months, the participants received different tennis learning and practical courses. The similarities and differences between EG and CG in the teaching methods and teaching goals are shown in Table 3 below. In short, apart from adding sports games to EG, EG, and CG were the same in each teaching stage and teaching content, and the overall teaching style followed a unified standard.

### 2.4. Sports Games

There are many different types of sports games. In the college physical education, tennis courses are an effective way to promote tennis. In this experimental teaching, the sports games for EG included three types: warm-up games, games for practicing a skill, and relaxing games. The sports games selected in this experimental teaching are shown in Table 4 below.

### 2.5. Data Analysis

Before the experimental teaching, each evaluation test item has undergone a normal test. SPSS 26.0 and Excel software were used as the tools. The T-test was conducted to evaluate the difference between EG and CG before and after the experimental teaching. The results of all test items were expressed in the form of mean ± standard deviation. When the significance level was *p* < 0.05, there was a significant difference between EG and CG. When the significance level reached *p* < 0.01, there was a very significant difference between EG and CG.

## 3. Results

### 3.1. Motivation

Before the experimental teaching, EG and CG have no significant difference in motivation evaluation variables, and the significance level *p* is above 0.05. Therefore, EG and CG were homogeneous initially before the experimental teaching. The details are shown in Table 5 below.

In this study, results of both groups’ tennis motivation questionnaires before and after the experimental teaching were compared. CG showed significant differences in motivation evaluation variables before and after the experimental teaching. Specifically, the two motivation evaluation variables, “vitality interest” and “active input” had significant differences and *p* < 0.05, while the other two motivation evaluation variables, “skill mastery” and “external encouragement” had no significant difference, and *p* > 0.05. This result shows that the traditional tennis training method has a stimulating ability on the motives of vitality interest and active input but no stimulating effect on skill mastery and external encouragement. The details are shown in Table 6 below.

Results of EG’s tennis motivation questionnaires before and after the experimental teaching were analyzed. EG showed significant differences in different motivation evaluation variables, among which “vitality interest” and “active input” showed very significant differences, and *p* < 0.01. This shows that sports games have a significant effect on students’ motivation to learn tennis. The details are shown in Table 7.

The tennis motivation questionnaires of EG and CG after the experimental teaching were compared. There were significant differences between EG and CG in several motivation evaluation variables after the experimental teaching. Among them, the active input was very significant, and *p* < 0.01. This result shows that experimental teaching has a great influence on students’ motivation to learn tennis. The motivation evaluation variables of students in EG are significantly better than those in CG. Therefore, the sports gamification method has a significant advantage in tennis training. The details are shown in Table 8 below.

### 3.2. Learning Performance

Before the experimental teaching, no significant differences were found in students’ tennis learning performance between EG and CG. The *p* values corresponding to each evaluation variable were above 0.05. This result shows the physical fitness and tennis skills of the participants in both groups before the experimental teaching are at the same level. The details are shown in Table 9 below.

CG’s mastery of tennis skills before and after the experimental teaching had significant differences. Specifically, students in CG had no significant differences in serve and footwork before and after the experimental teaching, but had very significant differences in forehand and backhand drives, *p* < 0.01. This result shows that students’ skills of forehand and backhand drives have been improved after the experimental teaching. The details are shown in Table 10 below.

EG’s mastery of tennis skills before and after the experimental teaching has significant differences. Specifically, the serve and footwork show significant differences, while the forehand and backhand drives show very significant differences. These results show that sports games have a significant impact on students’ performances in learning tennis skills. The evaluation results of each test item after experimental teaching are better than those before experimental teaching. This result shows that sports games have a significant impact on improving students’ tennis learning performance. The details are shown in Table 11 below.

After the experimental teaching, CG and EG have presented significant differences in learning tennis skills, as shown in Table 12 below.

## 4. Discussion

The above analysis of motivation and academic performance reveals the significant role of sports gamification in students’ tennis learning motivation, and sports games can play a vital role in improving students’ academic performance [24]. Based on the questionnaire of tennis classroom motivation, it is found that sports games are positive in improving students’ intrinsic motivation. The number of active inputs based on injective regulation and active interest based on intrinsic motivation increases most significantly, so the hypotheses 1 and 2 are verified. In the analysis of sports games and students’ mastery of tennis skills, it is found that sports games play a positive role in improving students’ tennis performance, and the difference between forehand and backhand drive is the most significant, so hypothesis 3 is confirmed. Generally, sports games in college tennis teaching positively promotes students’ sports ability, and stimulates students’ sports learning motivation and interest.

Lavega-Burgués and Navarro-Adelantado [25] made a comparative analysis on the integration of 117 games in sports, and considered the relevant sports dimension factors of sports internal logic and external institutional logic. They concluded that sports characteristics combined with games can positively improve the score of physical fitness, which is almost consistent with the results here. The motivation can be regarded as an internal logical component of physical education, while academic performance can be regarded as the result of external system. It is not difficult to find that sports gamification will have an impact on both of them, and this impact presents a promoting role. In addition, Santos Júnior, et al. [26] even revealed the positive role of sports games in improving the physical fitness of the elderly. The reason is that it is closely related to the characteristics of sports games. Sports games have various forms and are very interesting, which can arouse students’ learning enthusiasm and promote the concentration of attention. Wikman, et al. [27] revealed the importance of motivation for persistence by taking cricket as the research object, and found that the group with motivation intervention had higher intrinsic motivation and higher extrinsic motivation of self-determination. In view of the impact of educational gamification on students’ learning motivation, Chapman and Rich [28] investigated the role of gamification curriculum in stimulating students’ overall motivation through a cognitive survey. They found that 67.7% of the participants thought gamification curriculum was more incentive than traditional curriculum. This is basically consistent with the evaluation results obtained in this study. Menendez-Ferreira, et al. [29] even revealed the role of gamification technology in alleviating the occurrence of violence in the process of sports. This provides a solid support for the research work, and also a strong confirmation of the research results obtained.

In this experimental teaching, just as sports games affect students’ academic performance and motivation, in the process of sports game teaching, combined with the specific teaching situation and other factors, it is necessary to have a grasp of the whole teaching process systematically. The choice of sports game also needs to be based on the actual situation of students, and reasonable grouping and related risk factors should also be considered. Luo et al. [15] studied the relationship between sports games and learning motivation and skill level, and emphasized the important guiding role of teachers. However, in contrast, this study is more practical. Based on the situational game teaching mode, the research by Weidong, et al. [30] on physical education teaching revealed that the role of stimulus response selection and implementation in sports tactical decision-making, which proved part of the results obtained in this study, such as reasonable organization in the teaching process. Tsoy, et al. [31] in view of the research on the role of team games in teaching, revealed the positive role of game teaching in promoting people’s communication. Reasonable game links and content settings help to improve the relationship between people and promote team cooperation. In contrast, it is not difficult to find that the research results obtained are reasonable. Sports gamification positively affects the improvement of college students’ learning motivation and academic performance, and puts forward certain requirements for the flexibility and teamwork ability of the participants. It can play a positive role in improving learning interest, technical level, and teaching quality.

## 5. Conclusions

Tennis teaching is taken as the research object, and based on the experimental teaching method, the influence of sports gamification on students’ learning motivation and academic performance is discussed. The T-test on the differences between the experimental group and the control group before and after the experimental teaching suggests that, the adjustment of sports gamification on motivation can improve the participants’ academic performance and learning practice ability. Compared with CG using traditional teaching, EG has better academic performance and higher interest in tennis learning. Regarding the motivation, the number of active inputs based on introjection regulation and active interest based on intrinsic motivation increases more obviously, and the difference between forehand and backhand drive is the most significant. The research work provides a direction for the transformation and innovation of tennis teaching mode.

However, the motivation evaluation indicators and variables selected are not comprehensive enough. For instance, emotional factors including physiological and psychological state are not considered. In addition, the coverage of the selected samples is not enough, which is difficult to characterize the whole behavior. Therefore, in the future research, more incentive evaluation variables will be included and the research sample will be expanded to further reveal the internal relationship between gamification and sports.

## Figures and Tables

**Table 1:** Descriptive Data of Samples.

Groups	Gender	Number of Participants
CG	Male	60
	Female	50
	Total	110
EG	Male	50
	Female	40
	Total	90

Note: CG represents the control group of experimental teaching, and EG represents the experimental group of experimental teaching.

**Table 2:** Distribution of Experimental Teaching Stages.

Teaching Weeks	Teaching Contents
1st Week	<1> Basic theories; <2> Skills and tactics; <3> Rules of the game
2nd–5th Week	<1> Tennis grips; <2> Key point of movements for in-situ and moving forehand drive; <3> Footwork and forehand training
6–9th Week	<1> Key point of movements for the in-situ and moving backhand drive; <2> Footwork and backhand training; <3> Review of the forehand drive
10–12th Week	<1> Key point of movements for serving; <2> Serving training; <3> Review of forehand and backhand drives
13th Week	<1>; Consolidating the tennis skills; <2> Evaluation and tests

**Table 3:** Comparison of Teaching Methods and Goals between EG and CG.

Teaching Weeks	Teaching Methods		Teaching Goals	
	EG	CG	EG	CG
1st Week	The teachers explained the knowledge and asked questions. Students listened and answered.		Students had a general understanding of the basic situation of tennis, including theories, methods, and development status.	
2nd–5th Week	<1> Warm-up games; <2> Drive games;<3> Relaxing games	<1> The teacher explained and demonstrated; <2> Students copied and practiced; <3> The teacher corrected wrong movements	<1> Students mastered the essentials of griping and swinging a racket in forehand and backhand; <2> Students mastered the moving footwork and forehand and backhand drives; <3> Students actively participated in the games.	<1> Students mastered the essentials of griping and swinging a racket in forehand and backhand; <2> Students mastered the moving footwork and forehand and backhand drives; <3> Students mastered the key movements through group training.
6–9th Week				
10–12th Week	<1> Warm-up games; <2> Games of forehand and backhand drives; <3> Games to practice serving skills; <4> Relaxing games	<1> The teacher explained and demonstrated; <2> Students copied and practiced; <3> The teacher corrected the wrong movements	<1> Students mastered the serving skills; <2> Students actively participated in the games.	<1> Students mastered the serving skills; <2> Students mastered the key movements through group training.
13th Week		<1> Students addressed the gaps; <2> The teacher taught; <3> Students participated in the tests; <4> The teacher scored each student.	<1> Students strengthened the learned tennis skills; <2> Students learned the knowledge of competition skills and tactics, as well as basic knowledge of refereeing; <3> Students actively participated in the games; <4> Students took the tests according to the standard.	<1> Students consolidated and strengthened their basic skills; <2> Students learned the knowledge of competition skills and tactics, as well as basic knowledge of refereeing; <3> Students took the tests according to the standard.

Note: CG represents the control group of experimental teaching, and EG represents the experimental group of experimental teaching.

**Table 4:** Sports Games of Tennis Training.

Types of Sports Games	Names	Steps	Goals
Warm-up Games	Bouncing ball lightning round	Students were divided into several groups. The teacher shall ask questions, and students vied to answer the questions while bouncing the tennis ball with a racket. If every member of a group answered the question, the group won.	Reviewing the basic theories of tennis Improving the feeling Strengthening the ball-controlling technique
	Bouncing ball rally	Students were divided into several groups. Each member consecutively bounced the tennis ball, and the fastest group won.	Getting familiar with the racket and grips Enhancing team cooperation consciousness
	Tennis transportation	Students were divided into several groups. The teacher gave the order; four members of each group put the tennis ball in four places of the court consecutively. The fastest group won.	Getting familiar with the footwork Improving the moving skills Enhancing team cooperation and competition
	Bouncing ball front and back	The teacher gave the order. Students bounced the ball using the front and back of the racket. The winner bounced the ball more times within a particular period.	Improving the feeling Strengthening the ball-controlling technique
Games for Practicing a Skill	Barehand drive	Students were divided into two groups for competition. One of the groups threw the ball, mimicking forehand and backhand drive. The other group caught the ball and threw it back. Group members participated in the competition consecutively. The group with the least eliminated members won.	Improving the consistency of forehand and backhand drives Strengthening the essentials of drives
	Obstacle drive	Students were divided into several groups. Obstacles were set in the front of the player in a triangular shape so that the player must pass through the obstacles using correct footwork.	Enhancing the drive footwork Improving forehand and backhand skills
	Play catch	Students were divided into several groups. Within the groups, students were trained pairwise; one tossed the ball, and one caught the ball. Within a particular period, the pair with the most effective serve won. The final group winner depended on the points won by its pairs.	Improving the consistency of serve Strengthening the ball-controlling technique
	Big wheel	Five students were in a group. According to the circle behind the court baseline, four students stood on the north, south, east, and west sides, and the remaining student served the tennis ball before the baseline. Alternately, each group member served the ball ten times.	Strengthening footwork and mobility Promoting coordination of footwork movement and racket swing
Relaxing game	Rabbit dance	The group members stood in line, jumped, and raised their legs; the one who made a mistake was out. The group with the least eliminated members won.	Improving responsiveness Promoting coordination of limbs
	Numbered ball catching	Students stood in a circle and were assigned a number. One student stood in the center of the circle and threw the tennis ball while randomly calling out a number. The student with the corresponding number caught the ball. Those who made a mistake shall perform a show or be punished.	Strengthening the level of response and mobility

**Table 5:** Comparison of Motivation Evaluation Variables before Experiment Teaching.

Motivation Evaluation	EG (*n* = 75)	CG (*n* = 75)	*t*	*p*
Active Input	6.67 ± 2.75	6.72 ± 2.33	−0.05	0.966
Vitality Interest	5.86 ± 2.77	6.24 ± 2.72	−0.61	0.544
Skill Mastery	6.14 ± 3.34	6.51 ± 2.78	−0.05	0.586
External Encouragement	6.09 ± 2.99	6.01 ± 2.67	−0.12	0.907

Note: CG represents the control group of experimental teaching, and EG represents the experimental group of experimental teaching; the *t* value represents the result of the T-test, and *p* represents the significance level.

**Table 6:** Comparison of Motivation Evaluation Variables of CG before and after Experimental Teaching.

Motivation Evaluation	Before Experimental Teaching (*n* = 75)	After Experimental Teaching (*n* = 75)	*t*	*p*
Active Input	6.72 ± 2.33	7.52 ± 3.24	−2.31	0.028
Vitality Interest	6.24 ± 2.72	7.84 ± 3.76	−2.38	0.023
Skill Mastery	6.51 ± 2.78	7.02 ± 2.52	−0.42	0.683
External Encouragement	6.01 ± 2.67	7.51 ± 3.26	−0.58	0.567

**Table 7:** Comparison of Motivation Evaluation Variables of EG before and after Experimental Teaching.

Motivation Evaluation	Before Experimental Teaching (*n* = 75)	After Experimental Teaching (*n* = 75)	*t*	*p*
Active Input	6.67 ± 2.75	10.21 ± 3.33	−5.28	0.005
Vitality Interest	5.86 ± 2.77	9.44 ± 3.16	−4.52	0.004
Skill Mastery	6.14 ± 3.34	7.71 ± 3.39	−2.17	0.037
External Encouragement	6.09 ± 2.99	7.66 ± 2.43	2.07	0.046

**Table 8:** Comparison of Motivation Evaluation Variables of EG and CG after Experimental Teaching.

Motivation Evaluation	EG (*n* = 75)	CG (*n* = 75)	*t*	*p*
Active Input	10.21 ± 3.33	7.52 ± 3.24	−3.68	0.005
Vitality Interest	9.44 ± 3.16	7.84 ± 3.76	−2.07	0.041
Skill Mastery	7.71 ± 3.39	7.02 ± 2.52	−2.09	0.041
External Encouragement	7.66 ± 2.43	7.51 ± 3.26	2.46	0.017

Note: CG represents the control group of experimental teaching, and EG represents the experimental group of experimental teaching.

**Table 9:** Comparison of Tennis Learning Performance between EG and CG before Experimental Teaching.

Test items	EG (*n* = 75)	CG (*n* = 75)	*t*	*p*
Serve	25.16 ± 1.72	25.43 ± 2.18	0.618	0.538
Forehand Drive	5.82 ± 1.10	5.82 ± 0.63	−0.055	0.956
Backhand Drive	13.12 ± 3.18	12.92 ± 2.16	0.332	0.743
Footwork	15.38 ± 2.83	15.36 ± 2.84	0.074	0.944

Note: CG represents the control group of experimental teaching, and EG represents the experimental group of experimental teaching.

**Table 10:** Comparison of CG’s Tennis Learning Performance before and after Experimental Teaching.

Test Items	Before Experimental Teaching (*n* = 75)	After Experimental Teaching (*n* = 75)	*t*	*p*
Serve	25.43 ± 2.18	25.12 ± 1.68	0.74	0.466
Forehand Drive	5.82 ± 0.63	5.94 ± 0.76	−6.00	0.005
Backhand Drive	12.92 ± 2.16	18.96 ± 6.08	−7.07	0.004
Footwork	15.36 ± 2.84	18.62 ± 3.68	−0.06	0.957

**Table 11:** Comparison of EG’s Tennis Learning Performance before and after Experimental Teaching.

Test Items	Before Experimental Teaching (*n* = 75)	After Experimental Teaching (*n* = 75)	*t*	*p*
Serve	25.16 ± 1.72	24.16 ± 2.47	2.38	0.023
Forehand Drive	5.82 ± 1.10	5.99 ± 0.89	−1.84	0.007
Backhand Drive	13.12 ± 3.18	21.64 ± 4.39	−4.28	0.005
Footwork	15.38 ± 2.83	20.99 ± 3.86	−7.74	0.028

**Table 12:** Comparison of EG and CG based on Tennis Learning Performance after Experimental Teaching.

Test Items	EG (*n* = 75)	CG (*n* = 75)	*t*	*p*
Serve	24.16 ± 2.47	25.12 ± 1.68	2.38	0.023
Forehand Drive	5.99 ± 0.89	5.94 ± 0.76	−1.84	0.007
Backhand Drive	21.64 ± 4.39	18.96 ± 6.08	−4.28	0.005
Footwork	20.99 ± 3.86	18.62 ± 3.68	−7.74	0.028

Note: CG represents the control group of experimental teaching, and EG represents the experimental group of experimental teaching.

## Data Availability

The raw data supporting the conclusions of this article will be made available by the authors, without undue reservation.
